# Suppression of Poxvirus Replication by Resveratrol

**DOI:** 10.3389/fmicb.2017.02196

**Published:** 2017-11-17

**Authors:** Shuai Cao, Susan Realegeno, Anil Pant, Panayampalli S. Satheshkumar, Zhilong Yang

**Affiliations:** ^1^Division of Biology, Kansas State University, Manhattan, KS, United States; ^2^Poxvirus and Rabies Branch, Division of High-Consequence Pathogens and Pathology, National Center for Emerging and Zoonotic Infectious Diseases, Centers for Disease Control and Prevention, Atlanta, GA, United States

**Keywords:** poxvirus, vaccinia virus, monkeypox, resveratrol, DNA synthesis, gene expression, antiviral

## Abstract

Poxviruses continue to cause serious diseases even after eradication of the historically deadly infectious human disease, smallpox. Poxviruses are currently being developed as vaccine vectors and cancer therapeutic agents. Resveratrol is a natural polyphenol stilbenoid found in plants that has been shown to inhibit or enhance replication of a number of viruses, but the effect of resveratrol on poxvirus replication is unknown. In the present study, we found that resveratrol dramatically suppressed the replication of vaccinia virus (VACV), the prototypic member of poxviruses, in various cell types. Resveratrol also significantly reduced the replication of monkeypox virus, a zoonotic virus that is endemic in Western and Central Africa and causes human mortality. The inhibitory effect of resveratrol on poxviruses is independent of VACV N1 protein, a potential resveratrol binding target. Further experiments demonstrated that resveratrol had little effect on VACV early gene expression, while it suppressed VACV DNA synthesis, and subsequently post-replicative gene expression.

## Introduction

Smallpox is a deadly disease, responsible for approximately 300 million human deaths in the 20th century alone. Smallpox is caused by the variola virus, the most notorious member of the family *Poxviridae* (Miller et al., 2001). Despite the eradication of smallpox 37 years ago, poxviruses are of renewed interest due to their continuous impact on public health. Specifically, many poxviruses cause other human and animal diseases. For example, monkeypox, a zoonotic disease endemic in Central and Western Africa, caused an outbreak in humans in the United States (US) in 2003 (Reed et al., 2004; Bayer-Garner, 2005). Molluscum contagiosum accounts for 1 in 500 outpatient visits per year in the United States (Reynolds et al., 2009). Additionally, there is a concern that variola virus, the causative agent of smallpox, can potentially be used as a biological weapon from unsecured stocks or genetic engineering. Humans are particularly vulnerable to smallpox in the post-smallpox immunization era due to the absence of routine vaccination, waning immunity, and lower proportion of vaccinated individuals in the current population. In fact, between 1980 and 2010, the monkeypox incidence in Central Africa has increased 20 times after the discontinuation of smallpox immunization (Rimoin et al., 2010). In addition, poxviruses are developed as vectors for vaccine development against infectious diseases and as anti-cancer agents (Rerks-Ngarm et al., 2009; Draper and Heeney, 2010; Breitbach et al., 2011; Altenburg et al., 2014; Izzi et al., 2014). There are no FDA-approved drugs for poxvirus-infection treatment. Cidofovir, a drug for human cytomegalovirus infection, is an off-label drug to treat poxvirus infection (Robbins et al., 2005; Lu et al., 2011; Dower et al., 2012). There were also a number of small-molecule inhibitors of poxviruses identified in the past years, for example, CMX001, Tecovirimat (ST-246), and CMLDBU6128 (Quenelle et al., 2007; Huggins et al., 2009; Jordan et al., 2009). However, resistant viruses to the compounds were isolated in cell culture, including CMX001 and ST-246 (Yang et al., 2005; Andrei et al., 2006; Farlow et al., 2010). A combination therapy may be required to treat infected individuals, which demands the identification and characterization of additional poxvirus inhibitors.

Resveratrol is a natural polyphenol stilbenoid found in grapes, berries, and a number of other plants. Extensive studies have been carried out to investigate its functions in modulating lifespan, metabolism, cancer, and other diseases (Fremont, 2000). Resveratrol inhibits replication of a number of viruses, such as influenza virus, herpes simplex virus, enterovirus, hepatitis C virus, respiratory syncytial virus, human immunodeficiency virus, varicella zoster virus, Epstein-Barr virus, African swine fever virus, and duck enteritis virus (Docherty et al., 1999, 2006; Palamara et al., 2005; Nakamura et al., 2010; Galindo et al., 2011; Espinoza et al., 2012; Xie et al., 2012; Xu et al., 2013; Abba et al., 2015; Zhang et al., 2015). The antiviral mechanisms of resveratrol against these viral infections are diverse and include inhibition of viral protein synthesis, DNA synthesis, and modulation of host functions important for viral infection (Abba et al., 2015). In contrast to the above-mentioned viruses, resveratrol facilitates Kaposi’s-sarcoma associated herpesvirus (KSHV) reactivation from latency in several cell lines through enhancing mitochondrial function of infected cells (Yogev et al., 2014). Nevertheless, the effect of resveratrol on poxvirus replication has not been examined. A previous study showed that several polyphenols, including resveratrol, directly bind to and may inhibit vaccinia virus (VACV, the prototypic member of poxviruses)-encoded N1 protein, a cellular apoptotic regulator (Cheltsov et al., 2010). However, N1L is a non-essential gene and deletion of N1L from VACV genome does not affect VACV infection in cultured cells (Bartlett et al., 2002). Therefore, it is unlikely that resveratrol can prevent VACV infection through N1 protein in cell culture.

Here, we demonstrated that resveratrol could strongly suppress VACV replication in multiple cell types. We also showed that resveratrol directly targeted VACV DNA synthesis step and the suppression was independent of the viral N1 protein. Resveratrol also suppressed monkeypox virus (MPXV) replication.

## Materials and Methods

### Cell Culture

BS-C-1 cells (ATCC-CCL26) were cultured in Eagle’s Minimum Essential Medium (EMEM). HeLa cells (ATCC-CCL2) were cultured in Dulbecco’s Modified Eagle Medium (DMEM). Normal human dermal fibroblasts (NHDFs, ATCC PCS-201-010) and human foreskin fibroblasts (HFFs, kindly provided by Dr. Bernard Moss) were also cultured in DMEM. The EMEM and DMEM were supplemented with 10% fetal bovine serum (FBS), L-glutamine (2 mM), streptomycin (100 μg/mL), and penicillin (100 units/mL). Cells were cultured in an incubator with 5% CO_2_ at 37°C.

### Cell Viability Assay and Calculation of 50% Cytotoxicity Concentration (CC_50_)

HeLa cells and HFFs were cultured in 12-well plates. The cells were treated with DMSO or resveratrol at a series of concentrations. Cell viability was measured using trypan-blue exclusion test (Strober, 2015). After 24 h of treatment, cells in each well were treated with 300 μL of trypsin and resuspended with 500 μL of DMEM by pipetting. Twenty microliters of cell suspension was gently mixed with 20 μL of 4% trypan blue. The numbers of cells were measured with a hemocytometer. The CC_50_ was calculated using relative cell viability at different resveratrol concentrations by linear regression analysis.

### Viruses, Viral Infection, and Titration

Vaccinia virus Western Reserve (WR, ATCC VR-1354) strain was amplified and purified as described previously (Earl et al., 2001a). Recombinant N1L-deleted VACV was generated by homologous recombination and the N1L gene was replaced with a green fluorescent protein (GFP) gene. Briefly, PCR product of GFP coding sequence under a late P11 promoter flanked by 500-bp homologous sequences upstream and downstream N1L gene was transfected into VACV-infected HeLa cells. The transfected cells were collected at 24 h post-infection (hpi). Recombinant viruses expressing GFP were clonally purified by multiple rounds of plaque isolation (Earl et al., 2001b). Recombinant VACV with the correct insertion or deletion was verified by PCR. The recombinant VACV that expresses GFP under a synthetic early/late VACV promoter (Chakrabarti et al., 1997) and dsRED under P11 VACV promoter was generated using a similar procedure. Recombinant virus vP11-Fluc that expresses firefly luciferase gene under the late VACV P11 promoter was described elsewhere (Bengali et al., 2011). MPXV MPXV-WA 2003-044 and MPXV-ROC 2003-358 clades were utilized in this study. Preparation, infection, and titration of VACV and MPXV were carried out as described previously (Earl et al., 2001a). For infection, cells were incubated with desired amount of viruses in DMEM (containing 2.5% FBS). After 1 h of incubation at 37°C in 5% CO_2_, virus-containing DMEM was replaced with fresh DMEM (containing 2.5% FBS) and further incubated for desired amount of time. For titration, BS-C-1 cells cultured in 6- or 12-well plates were infected with serial diluted viral samples and incubated in DMEM (containing 2.5% FBS and 0.5% methyl cellulose) for 48 h. The cells were stained with 0.1% crystal violet for 5 min and washed with water before counting the number of plaques.

### Measurement and Calculation of 50% Inhibiting Concentration (IC_50_)

HeLa cells or HFFs were cultured in 12-well plates. The cells were infected with VACV at a multiplicity of infection (MOI) of 1 in the presence of DMSO or resveratrol at a series of concentrations. After 24 hpi, virus titers were measured by a plaque assay. The IC_50_ was calculated using virus inhibitory efficiency at different resveratrol concentrations by linear regression analysis.

### Antibodies and Chemical Inhibitors

Antibodies against VACV L2 protein, P4a (A10) protein, and whole VACV viral particle were kindly provided by Dr. Bernard Moss. Antibody against human GAPDH was purchased from Abcam (Cambridge, MA, United States). Chemicals cytosine-1-β-D-arabinofuranoside (AraC), resveratrol, and hydroxyurea were purchased from Sigma (St. Louis, MO, United States).

### Western Blotting Analysis

Cells were collected and lysed in NP-40 cell lysis buffer (150 mM NaCl, 1% NP-40, 50 mM Tris–Cl, pH 8.0). Cell lysates were reduced by 100 mM DTT and denatured by sodium dodecyl sulfate–polyacrylamide gel electrophoresis (SDS–PAGE) loading buffer and boiling for 3 min before SDS–PAGE, followed by transferring to a polyvinylidene difluoride membrane. The membrane was then blocked in TBS-Tween (TBST) [50 mM Tris–HCl (pH 7.5), 200 mM NaCl, 0.05% Tween 20] containing 5% skim milk and 1% bovine serum albumin for 1 h, incubated with primary antibody in the same TBST-milk buffer for 1 h, washed with TBST three times for 10 min each time, incubated with horseradish peroxidase-conjugated secondary antibody for 1 h, washed three times with TBST, and developed with chemiluminescent substrate (National Diagnostics, Atlanta, GA, United States). The whole procedure was carried out at room temperature. Antibodies were stripped from the membrane by Restore (Thermo Fisher Scientific, Waltham, MA, United States) for western blot analysis using another antibody.

### Luciferase Assay

Firefly luciferase activities were measured by an ENSPIRE plate reader (PerkinElmer, Waltham, MA, United States) using the Luciferase Assay System (Promega, Madison, WI, United States) according to manufacturer’s instructions.

### Plasmid Replication in VACV-Infected Cells

Total DNA was isolated using E.Z.N.A.^®^ Blood DNA Kit (Omega Bio-Tek, Inc., Norcross, GA, United States). One microgram of DNA was treated with a DpnI enzyme to digest originally transfected input plasmid DNA (amplified from *Escherichia coli*, with methylation on DpnI recognition site) but not the plasmid DNA amplified in mammalian cells (no methylation in DpnI site). The plasmid DNA amounts were then measured using qPCR using a pair of primers that amplify a fragment containing a DpnI site.

### Quantitative Real-Time PCR

Total DNA was extracted from mock- or VACV-infected cells at indicated time points using E.Z.N.A.^®^ Blood DNA Kit. Relative viral DNA levels were quantified by CFX96 real-time PCR instrument (Bio-Rad, Hercules, CA, United States) with All-in-one^TM^ 2× qPCR mix (GeneCopoeia) and primers specific for VACV and human genomes, respectively. The qPCR program was started with initial denaturation step at 95°C for 3 min, followed by 40 cycles of denaturation at 95°C for 10 s, annealing and reading fluorescence at 52°C for 30 s, and extension at 72°C for 30 s. The primers used in this study are:

C11pF: AAACACACACTGAGAAACAGCATAAA;
C11pR: ACTATCGGCGAATGATCTGATTATC;
GAPDH-F: ACATCAAGAAGGTGGTGAAGCA;
GAPDH-R: CTTGACAAAGTGGTCGTTGAGG. The primers used for recombinant N1L-deletion VACV characterization are:
N1-F: TTATTTTTCACCATATAGATCAATCATTAGATCAT.
N1-R: ATGAGGACTCTACTTATTAGATATATTCTTTGGAG.
Puc19-F: TGCGCGTAATCTGCTGCTTG.
Puc19-R: CGAGGTATGTAGGCGGTGCT.

### Statistical Analysis

All titration data were represented as the means of at least three independent experiments. One-tailed paired *T*-test was used to access for significant difference between two means with *P* < 0.05.

## Results

### Resveratrol Suppresses VACV Replication in Immortal and Primary Human Cells

**FIGURE 1:**
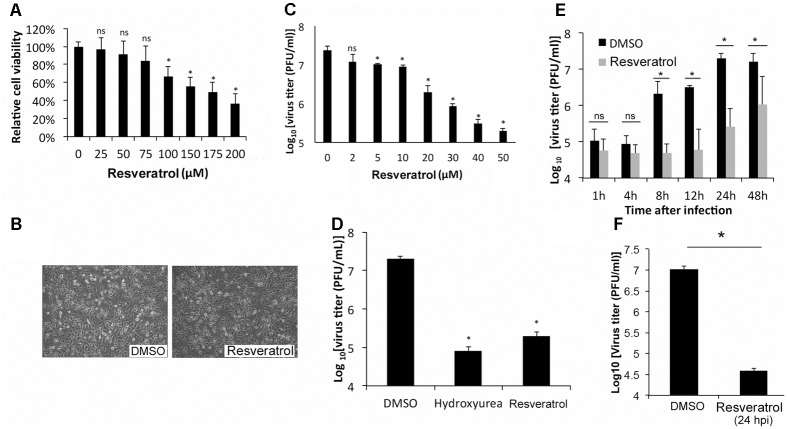
Resveratrol suppresses VACV replication in HeLa cells. **(A)** HeLa cells were treated with DMSO or resveratrol at the indicated concentrations for 24 h. Cell viability was measured using trypan blue exclusion test. **(B)** HeLa cells were imaged under the bright field of a microscope after DMSO or resveratrol (50 μM) treatment for 24 h. **(C)** HeLa cells were infected with VACV at an MOI of 1 in the presence of resveratrol at the indicated concentrations. Virus yield at 24 hpi were determined by a plaque assay. **(D)** HeLa cells were infected with VACV at an MOI of 1 in the presence of DMSO, hydroxyurea (10 mM), or resveratrol (50 μM). Virus yield at 24 hpi were determined by a plaque assay. **(E)** HeLa cells were infected with VACV at an MOI of 0.01 in the presence of DMSO or 50 μM resveratrol. Virus titers were determined by a plaque assay at the indicated time points. **(F)** HeLa cells were infected with VACV at an MOI of 0.001 treated with DMSO or 50 μM resveratrol at 24 hpi. Virus titers were determined by a plaque assay at 72 hpi. The asterisk indicates significant difference (*P* < 0.05) and the ns indicates no significant difference between DMSO-treated cells and resveratrol- or hydroxyurea-treated cells. The error bar indicates standard deviation.

To test the effect of resveratrol on the viability, HeLa cells, an immortal cervical cancer cell line (Scherer et al., 1953), were treated at a series of concentrations. Cell viability assay showed that resveratrol caused 50% HeLa cell death (CC_50_) at the concentrations of 157.75 μM in 24 h (**Figure 1A** and **Table 1**). Consistent with the result, no significant morphological change was observed for HeLa cells at the concentration of 50 μM (**Figure 1B**). We then examined the effect of resveratrol on VACV replication. HeLa cells were infected with VACV at an MOI of 1 in the presence of a series of concentrations of resveratrol and the viral titers were measured 24 hpi. The concentration of resveratrol that resulted in 50% inhibition (IC_50_) of VACV replication was 4.72 μM (**Figure 1C** and **Table 1**). Resveratrol reduced virus yield by more than 120-fold at the concentration of 50 μM (**Figure 1C**). The inhibitory effect of VACV replication by resveratrol is comparable to a well-characterized VACV inhibitor, hydroxyurea, which is known to prevent VACV DNA synthesis and decreased virus yield by approximately 200-fold at the concentration of 10 mM under the same infection conditions (**Figure 1D**). We examined the effect of resveratrol on multiple rounds of VACV replication by infecting HeLa cells at a low MOI of 0.01 and measuring the viral yield at different times post VACV infection. We observed significant reduction of viral titers in resveratrol-treated cells started from 8 hpi (**Figure 1E**), again demonstrating that resveratrol severely impaired the replication of VACV in HeLa cells. Moreover, the addition of resveratrol at 24 hpi still reduced VACV replication by 250-fold when the initial MOI is low (0.001) (**Figure 1F**), suggesting a possible use of resveratrol to prevent viral spreading post infection.

**Table 1:** Inhibitory effect of resveratrol on VACV replication and cytotoxicity.

Cells	IC_50_ (μM)^a^	CC_50_ (μM)^b^
HFF	3.51 ± 1.22	176.88 ± 17.44
HeLa	4.72 ± 2.34	157.75 ± 23.66

^a^*The concentration of resveratrol that reduces the yield of VACV by 50%. ^b^The concentration of resveratrol that causes 50% cell death*.

**FIGURE 2:**
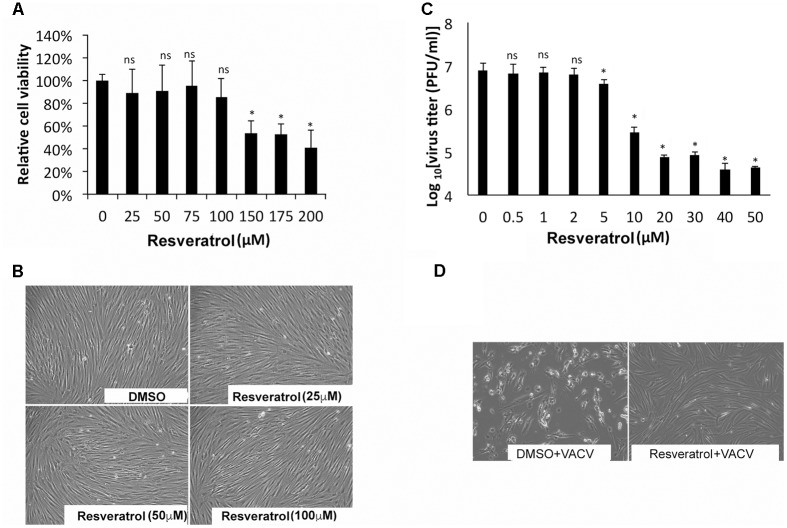
Resveratrol suppresses VACV replication in HFFs. **(A)** HFFs were treated with DMSO or resveratrol at the indicated concentrations for 24 h. Cell viability was measured using trypan blue exclusion test. **(B)** DMSO- or resveratrol-treated cells were imaged under the bright field of a microscope. **(C)** HFFs were infected with VACV at an MOI of 1 in the presence of resveratrol at the indicated concentrations. Virus yield at 24 hpi was determined by a plaque assay. **(D)** HFFs were infected with VACV at an MOI of 0.01 in the presence of resveratrol at the indicated concentrations. At 72 hpi, cells were imaged under the bright field of a microscope. The asterisk indicates significant difference (*P* < 0.05) and the ns indicates no significant difference between DMSO-treated cells and resveratrol-treated cells. The error bar indicates standard deviation.

The effect of resveratrol on VACV replication in primary human cells such as HFFs was also tested. The CC_50_ concentration of resveratrol on HFF was 176.88 μM (**Figure 2A** and **Table 1**). In fact, at the concentration of up to 100 μM, resveratrol did not affect the morphology of HFFs (**Figure 2B**). The IC_50_ concentration of resveratrol in HFFs was 3.51 μM and the virus yield of VACV from 50 μM resveratrol-treated HFFs was reduced by approximately 200-fold at an MOI of 1 (**Figure 2C** and **Table 1**). Moreover, treatment of HFFs with 100 μM of resveratrol protected the HFFs from VACV infection-induced cytopathic effects of the cells (**Figure 2D**). In addition, resveratrol also reduced the replication of VACV in another primary human cell type, NHDF (not shown). Taken together, our results demonstrate that resveratrol dramatically reduces VACV replication in different human cell types.

### Resveratrol Suppresses MPXV Replication

**FIGURE 3:**
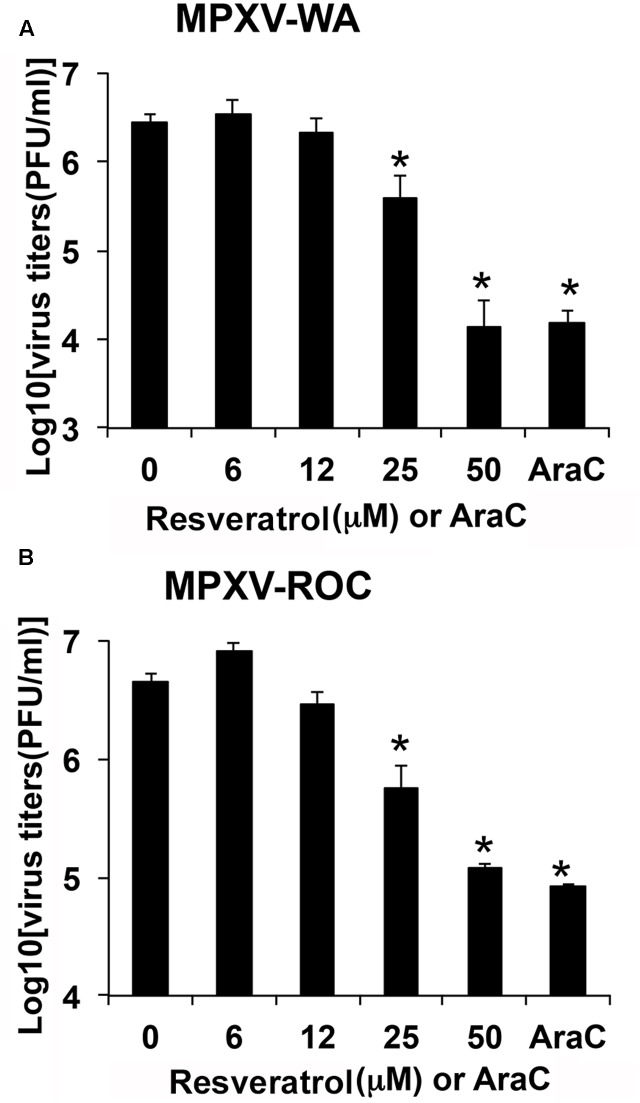
Resveratrol suppresses monkeypox infection. HeLa cells were infected with MPXV-WA **(A)** or MPXV-ROC **(B)** at an MOI of 1 in the presence of resveratrol at the indicated concentrations or AraC (40 μg/mL). Virus yield at 24 hpi was determined by a plaque assay. The asterisk indicates significant difference (*P* < 0.05) between DMSO-treated cells and resveratrol- or AraC-treated cells. The error bar indicates standard deviation.

We examined the effect of resveratrol on MPXV replication. HeLa cells were infected with MPXV-WA and MPXV-ROC, respectively, at an MOI of 1 in the presence of a series of concentrations of resveratrol and the viral titers were measured 24 hpi. As shown in **Figures 3A,B**, 50 μM resveratrol reduced the virus yield of MPXV-WA and MPXV-ROC clades by 195- and 38-fold, respectively. The IC_50_ was 12.41 μM for WA strain and 15.23 μM for ROC strain (**Table 2**). The inhibitory effect of MPXV replication by resveratrol was comparable to the well-characterized orthopoxvirus (OPXV) inhibitor, AraC, in the corresponding parallel experiments (**Figure 3**).

**Table 2:** Inhibitory effect of resveratrol on MPXV replication in HeLa cells.

MPXV strains	IC_50_ (μM)^a^
WA	12.41 ± 3.28
ROC	15.23 ± 2.71

^a^*The concentration of resveratrol that reduces the yield of MPXV by 50%*.

### Resveratrol Suppresses N1L-Deleted VACV Replication

**FIGURE 4:**
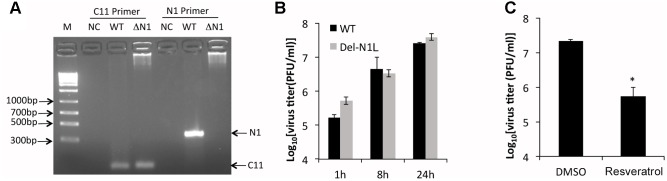
Resveratrol suppresses N1L-deleted VACV replication. **(A)** Deletion of N1L gene from recombinant virus was confirmed by PCR with two pairs of primer specific for C11R gene (positive control) and N1L gene, respectively. **(B)** HeLa cells were infected with wild type and N1L-deleted VACV at an MOI of 1. Virus growth was determined by a plaque assay. **(C)** HeLa cells were infected with N1L-deleted VACV at an MOI of 1 in the presence or absence of resveratrol (50 μM). Virus titers were determined by a plaque assay at 24 hpi. The asterisk indicates significant difference (*P* < 0.05). The error bar indicates standard deviation.

N1L encodes a viral virulence factor that is expressed at early stage of VACV gene expression and regulates host cell apoptosis (Bartlett et al., 2002; Yang et al., 2010). It has been reported that some polyphenols, including resveratrol, could directly bind to and may inhibit the function of N1 protein (Cheltsov et al., 2010). The authors further speculated that resveratrol might inhibit VACV replication by targeting the N1 protein. However, the effect of resveratrol on VACV replication was not tested in the aforementioned study. Moreover, the N1L is not an essential VACV gene and the deletion of N1L from VACV genome was not shown to affect VACV replication in cultured cells (Bartlett et al., 2002). Based on these facts, we reasoned that prevention of VACV replication by resveratrol is not through the N1 protein. To test it, we replaced the N1L gene with a GFP gene in the VACV genome through homologous recombination (**Figure 4A**). Consistent with a previous observation (Bartlett et al., 2002), the deletion of N1L did not affect VACV replication and viral yields (**Figure 4B**). As expected, resveratrol similarly suppressed VACV-Del-N1L virus (**Figure 4C**), indicating that inhibitory effect is not mediated through the N1 protein.

### Resveratrol Suppresses VACV Late, But Not Early Gene Expression

**FIGURE 5:**
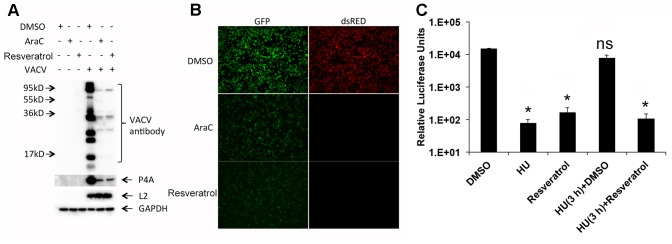
Resveratrol suppresses VACV late, but not early gene expression. **(A)** HeLa cells were infected with VACV at an MOI of 1 in the presence of DMSO, AraC (40 μg/mL), or resveratrol (50 μM). At 24 hpi, the expression of viral proteins in infected cells was detected by western blotting using the indicated antibodies. **(B)** HeLa cells were infected with a recombinant VACV carrying a GFP gene under an early/late VACV promoter and a dsRED gene under a late VACV promoter at an MOI of 1 in the presence of DMSO, AraC (40 μg/mL), or resveratrol (50 μM). The expression of GFP and dsRED was observed and imaged using a fluorescent microscope. **(C)** HeLa cells were infected with VACV-P11-Fluc at an MOI of 1 and treated with hydroxyurea (HU, 10 mM) from 0 to 3 hpi. Then hydroxyurea-containing medium was washed away and replaced with cell culture medium containing DMSO or resveratrol (50 μM), and further incubated for another 3 h until luciferase activity in the infected cell lysates was measured. Luciferase activities from infected cells treated with only DMSO, hydroxyurea, or resveratrol through 0–6 hpi were also measured. The asterisk indicates significant difference (*P* < 0.05) between control and treated cells. The ns indicates no significant difference. The error bar indicates standard deviation.

To investigate the stage of viral life cycle targeted by resveratrol, we examined the effect of resveratrol treatment on VACV protein expression by Western blot analysis (**Figure 5A**). Anti-VACV serum was derived from rabbits immunized with purified VACV particles that comprise mostly viral structural proteins expressed at the late stage of VACV gene expression. P4a is a major viral core protein encoded by the VACV late gene A10L (Yang et al., 2011). L2 protein is involved in VACV morphogenesis and is expressed at the early stage of VACV gene expression (Yang et al., 2010; Maruri-Avidal et al., 2011a,b). DNA synthesis inhibitor AraC was used as a positive control. Western blots with anti-VACV serum and P4a antibodies demonstrated dramatic reduction in protein levels in the presence of resveratrol and at levels comparable to the AraC treatment. In contrast, both resveratrol and AraC treatments did not affect the expression level of the viral early protein L2 (**Figure 5A**). We also used a recombinant VACV that expressed GFP under an early/late VACV promoter and dsRED under a late VACV promoter to confirm suppression of late protein synthesis by resveratrol. HeLa cells infected with recombinant VACV expressing fluorescent proteins at an MOI of 1 in the presence of resveratrol, AraC, or vehicle control DMSO were observed under a fluorescent microscope. The results clearly showed that both resveratrol and AraC completely blocked dsRED expression that was expressed at the late stage of gene expression, while they only partially suppressed GFP expression at similar levels that could also be expressed at the early stage of VACV replication (**Figure 5B**). These results indicate that resveratrol has little or only moderate effect on VACV replication prior to viral early gene expression but affects a replication step between the early and late stages of gene expression.

The third approach we employed to examine the effect of resveratrol on VACV late gene expression was using a combination of hydroxyurea and resveratrol. Hydroxyurea blocks VACV DNA synthesis but not early gene expression (Katz et al., 1974). In the control experiment, hydroxyurea and resveratrol were confirmed for their inhibitory effects on expression of VACV late promoter-controlled firefly luciferase gene of vP11-Fluc in HeLa cells (**Figure 5C**). In the parallel experiment, HeLa cells were infected with vP11-Fluc for 3 h in the presence of hydroxyurea, which allowed early gene expression. The hydroxyurea was washed away and DMSO or resveratrol was added and incubated for an additional 3 h. As can be seen, resveratrol still reduced luciferase activity while DMSO could not (**Figure 5C**).

Together, these results indicated that resveratrol affected a post-replication step after VACV early gene expression.

### Resveratrol Interferes VACV DNA Synthesis

**FIGURE 6:**
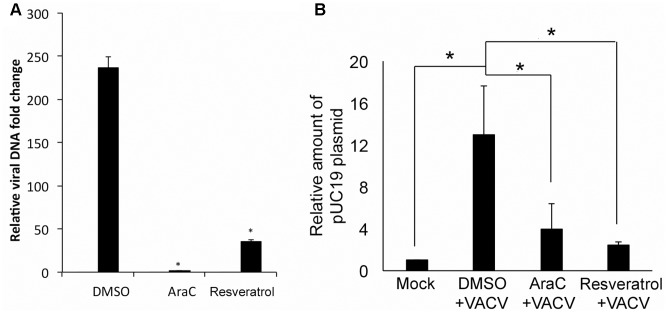
Resveratrol suppresses VACV DNA synthesis. **(A)** HeLa cells were infected with VACV at an MOI of 1 in the presence of DMSO, AraC (40 μg/mL), or resveratrol (50 μM). Relative viral DNA levels in infected cells were determined by real-time PCR at 1 and 24 hpi. The viral DNA level at 24 hpi was determined as the fold to the viral DNA level at 1 hpi. **(B)** HeLa cells were transfected with 200 ng of pUC19 plasmid and incubated overnight. The cells were then infected with VACV at an MOI of 5 or mock-infected in the presence of AraC, resveratrol, or DMSO. Total DNA was extracted from the cells at 24 hpi and 1 μg of total DNA was digested with DpnI at 37°C for 2 h followed by real-time qPCR using primers amplifying pUC19 fragment containing DpnI digestion site. The asterisk indicates significant difference (*P* < 0.05) and the ns indicates no significant difference between 1 and 24 hpi. The error bar indicates standard deviation.

The effect of resveratrol on VACV DNA synthesis was investigated since it is essential for post-replicative gene expression (intermediate and late protein synthesis). VACV DNA synthesis starts between 2 and 4 hpi in infected HeLa cells under the conditions used in this study (Yang et al., 2010). We examined VACV DNA amounts in VACV-infected HeLa cells at 1 and 24 hpi using quantitative real-time PCR (AraC was used as positive control). Our results indicated that resveratrol treatment significantly reduced VACV DNA amount at 24 hpi (**Figure 6A**). The VACV DNA was 237-fold higher in DMSO-treated cells, while the viral DNA amounts only increased 35- and 6-fold in resveratrol- and AraC-treated cells, respectively (**Figure 6A**).

We tested the direct inhibitory effect on DNA synthesis by resveratrol through examining plasmid DNA synthesis in VACV-infected cells as circular DNA can be replicated in VACV-infected cells that require all known viral proteins needed for VACV DNA synthesis (DeLange and McFadden, 1986; De Silva and Moss, 2005). We transfected pUC19 plasmid into HeLa cells for 12 h and then infected with VACV or mock-infected in the presence or absence of resveratrol and AraC. Total DNA was isolated from cells at 24 hpi and treated with DpnI that only digests methylated input DNA. The DNA was then measured using specific primers amplifying a pUC19 fragment containing the DpnI digestion site. Dpn-resistant plasmid DNA increased 12-fold in VACV-infected cells compared to DMSO treatment. However, there was only a 2- to 3-fold increase of DpnI-resistant plasmid DNA in VACV-infected cells treated with resveratrol or AraC treatment (**Figure 6B**). This result indicated that resveratrol could interfere viral DNA synthesis directly in VACV-infected cells.

## Discussion

Our study, for the first time, demonstrated a strong suppressive effect of resveratrol on poxvirus replication. Similar to other viruses, VACV replication is generally divided into entry, gene expression, genome replication, viral particle assembly, and exit steps. VACV gene expression is programmed as a cascade to express viral genes at early, intermediate, and late stages (Moss, 2013a). The early gene expression starts immediately after VACV enters into the infected cells, as the viral infectious particles package all the factors and enzymes needed for early viral mRNA synthesis. The viral early gene products include those necessary factors for viral DNA synthesis. The VACV DNA synthesis is required for viral intermediate, and subsequently, late gene expression. The intermediate and late gene products comprise most of the structural proteins to build infectious viral particles (Moss, 2013a). Our study indicates that the resveratrol directly targets viral DNA synthesis step to prevent VACV replication. Genome uncoating is a step needed to expose encapsidated viral DNA as a template for DNA synthesis. Because resveratrol does not block synthesis of viral early proteins and the viral genome uncoating factor D5 is an early protein (Kilcher et al., 2014), it is unlikely that resveratrol can prevent poxvirus genome uncoating. However, we do not completely rule out the possibility that resveratrol interferes poxvirus genome uncoating to some extent. Interestingly, the effect is independent of the non-essential viral N1L gene, albeit resveratrol has been suggested to be an inhibitor of the VACV N1 protein (Cheltsov et al., 2010).

As the prototypic member of poxvirus family, VACV has a linear, double-stranded DNA genome that replicates entirely in the cytoplasm (Moss, 2013a). The size of the genome is approximately 200 kbp. Although the molecular mechanism involved in VACV DNA synthesis is not fully understood, it is known that the VACV genome encodes most proteins required for replicating its DNA genome (Moss, 2013b). These proteins include a DNA polymerase encoded by E9L gene (Jones and Moss, 1984; Traktman et al., 1984), a helicase–primase encoded by D5R (Roseman and Hruby, 1987), a processivity factor encoded by A20R (McDonald et al., 1997), a uracil DNA glycosylase encoded by D4R (Upton et al., 1993), and a few other proteins bearing different roles in copying the viral DNA (Moss, 2013b). It has been shown that resveratrol inhibits multiple mammalian DNA polymerases including polymerase alpha through its 4-hydroxystyryl moiety, subsequently suppressing active DNA synthesis (Locatelli et al., 2005). As VACV DNA polymerase has considerable similarity to human polymerase alpha (Wang et al., 1989), it is highly possible that resveratrol interferes with VACV DNA polymerase activity directly. Resveratrol also modulates numerous cellular functions (Fremont, 2000); therefore, it is possible that resveratrol affects a cellular function that is important for VACV genome replication. However, the role of cellular functions in VACV DNA synthesis is poorly understood; thus, it is difficult to have an educated prediction of a specific cellular function that may be involved in this process.

All steps of VACV replication, from viral entry and exit, may be targeted for antiviral drug development. For example, mitoxantrone blocks VACV replication by targeting the virion assembly step (Deng et al., 2007). However, the viral DNA synthesis is one of the major targets for anti-poxvirus drug development. Several compounds that are used to treat poxvirus infection target the viral DNA synthesis step. Cidofovir, an acyclic nucleoside that is approved to treat cytomegalovirus infection in AIDS patients also exhibits anti-poxvirus activity by targeting DNA synthesis (Andrei and Snoeck, 2010). The widely used poxvirus inhibitors, AraC, hydroxyurea, and a recently identified inhibitor, CMX001, also target VACV DNA synthesis (Quenelle et al., 2007). The identification of resveratrol as a VACV DNA synthesis inhibitor may allow for developing alternative or compensative strategies to better manage current and re-emergent poxvirus infections and complications caused by poxviruses-based therapeutics.

## Conclusion

We showed that resveratrol, a member of natural plant polyphenols that is under extensive investigation of its effects on many biological processes, dramatically reduced VACV and MPXV replication. The suppression appears to affect the viral DNA synthesis step. The results will prompt further investigation of its effect on other poxvirus replication steps as well as the mechanism to inhibit VACV replication.

## Author Contributions

ZY, PS, and SC contributed to the conception of the study. SC, SR, and AP performed the experiments. SC and SR analyzed the data. ZY, SC, and PS wrote the manuscript.

## Conflict of Interest Statement

The authors declare that the research was conducted in the absence of any commercial or financial relationships that could be construed as a potential conflict of interest. The reviewer IAB and handling Editor declared their shared affiliation.
